# The ethics of talking about ‘HIV cure’

**DOI:** 10.1186/s12910-015-0013-0

**Published:** 2015-03-27

**Authors:** Stuart Rennie, Mark Siedner, Joseph D Tucker, Keymanthri Moodley

**Affiliations:** Center for Bioethics, Department of Social Medicine, University of North Carolina at Chapel Hill, Chapel Hill, USA; Department of Medicine, Massachusetts General Hospital and Harvard Medical School, Boston, USA; Institute for Global Health and Infectious Diseases, University of North Carolina at Chapel Hill, Chapel Hill, USA; UNC Project-China, Guangdong Provincial Dermatology/STD Hospital, Guangzhou, China; Center for Medical Ethics and Law, Department of Medicine, Stellenbosch University, Tygerberg, South Africa

**Keywords:** HIV/AIDS, Ethics, HIV cure, Biomedical research

## Abstract

**Background:**

In 2008, researchers reported that Timothy Brown (the ‘Berlin Patient’), a man with HIV infection and leukemia, received a stem-cell transplant that removed HIV from his body as far as can be detected. In 2013, an infant born with HIV infection received anti-retroviral treatment shortly after birth, but was then lost to the health care system for the next six months. When tested for HIV upon return, the child (the ‘Mississippi Baby’) had no detectable viral load despite cessation of treatment. These remarkable clinical developments have helped reinvigorate the field of ‘HIV cure’ research.

**Discussion:**

Although this research field is largely in a pre-clinical phase, talk about curing HIV has become a regular feature in the global mass media. This paper explores the language of HIV cure from philosophical, ethical and historical perspectives. Examination of currently influential definitions of ‘functional’ and ‘sterilizing’ HIV cure reveal that these conceptualizations are more complicated than they seem. Cure is often understood in narrowly biomedical terms in isolation from the social and psychological dimensions of illness. Contemporary notions of HIV cure also inherit some of the epistemic problems traditionally associated with cures for other health conditions, such as cancer. Efforts to gain greater conceptual clarity about cure lead to the normative question of how ‘HIV cure research’ ought to be talked about.

**Summary:**

We argue that attention to basic concepts ethically matter in this context, and identify advantages as well as potential pitfalls of how different HIV/AIDS stakeholders may make use of the concept of cure. While concepts other than cure (such as remission) may be appropriate in clinical contexts, use of the word cure may be justified for other important purposes in the struggle against HIV/AIDS.

*Mankind is extremely fond of every thing that promises a sudden or miraculous cure.*

William Buchan

*Domestic Medicine: Or a Treatise on the Prevention and Cure of Diseases by Regime and Simple Medicines (1871).*

## Background

### The rise of HIV cure language

The incurability of HIV is an important aspect of its social history. At the start of the epidemic, HIV was the plague of gay men and junkies, frighteningly stigmatized behaviors, and socially problematic modes of transmissibility. People with HIV had observable physical wasting and co-infections, and there were no effective treatments. Almost every person infected with the virus developed AIDS and died. When and where antiretroviral therapy (ART) has become available, policies, public perceptions, and personal experiences have largely shifted as HIV is viewed less as a death sentence, and more as a treatable, chronic condition. Another salient new shift may be on the horizon. A number of recent developments have, in different ways, provided hope that HIV could be controlled more comprehensively than thought possible in the past:

- *Bone marrow transplantation*. Timothy Brown received a bone marrow transplant using cells from a donor with a rare genetic mutation that confers resistance to HIV infection. Twenty months after the procedure, researchers reported they could find no trace of HIV in the recipient’s bone marrow, blood or other organ tissues [1]. Three patients in Boston that underwent a marrow transplantation procedure – albeit without the genetic mutation that confers resistance -- initially seemed to attain similar results, but subsequently experienced relapse [2].
- *Initiation of ART in acute infection: adults*. The French National Agency of AIDS Research (ANRS) collected data on HIV-infected adults in France who initiated ART early after HIV infection. A recent report described the medical course of 14 patients (the ‘Visconti cohort’) who started ART within ten weeks of infection, and were on treatment for an average of 3 years. After stopping treatment, the majority had undetectable viral loads for an average of 7.5 years [3]. Researchers estimated that the probability of maintaining viral control at 24 months after discontinuing treatment was only 15%, but the cohort may hold important clues about a possible cure for HIV.
- *Initiation of ART in acute infection: infants*. An infant diagnosed with HIV infection (the Mississippi Baby’) at 30 hours after birth received aggressive ART until 18 months of age. The infant was then lost to the healthcare system for the next 6 months. When tested for HIV upon return, the child had no detectable viral load despite cessation of treatment [4]. Unfortunately, after two medication-free years HIV was detected in the child’s blood and antiretroviral therapy was initiated [5]. A second infant treated early with ART (the ‘Long Beach Baby’) achieved similar results, but with an important difference: the child is still on ART [6]. In Canada, in the light of the Mississippi Baby case, researchers identified a cohort of HIV-infected infants that had been started on aggressive ART shortly after birth [7]. Treatment of one of the infants was interrupted after 3 years and 3 months, but unfortunately after two weeks the child’s viral load increased dramatically and ART was resumed [8].

How were these developments described? In the scientific literature, the ‘Berlin Patient’ was described as a case of ‘long-term control of HIV’ (Hutter G, Nowak D, Mossner M, et. al. [1]). The article reporting the ‘Visconti cohort’ in France similarly described the patients’ condition in terms of ‘long-term immunovirologic control’ (Saez-Cirion A, Bacchus C, Hocqueloux L et. al. [9]). In the New England Journal of Medicine, the ‘Mississippi Baby’ case was reported in terms of ‘functional cure’ and ‘absence of detectable viremia’ (Persaud D, Gay H, Ziemniak C, et. al. [10]). Press reports, however, overwhelmingly chose to describe these same cases in terms of actual or potential HIV *cure.*

### What does cure mean? Sterilizing and functional cures

What does ‘cure’ mean in HIV cure research? The scientific HIV cure literature makes regular reference to different kinds of cures, and definitions have been offered. The Food and Drug Administration (FDA) broadly defines HIV cure research as an investigation evaluating therapeutic interventions that would control or eliminate HIV infection to the point where no treatment would be needed to maintain health [11]. According to the International AIDS Society (IAS), a sterilizing HIV cure means: the elimination of all HIV-infected cells; a functional HIV cure means the life-long control of virus in the absence of ART, without achieving complete eradication of HIV. More specifically, the IAS sees the achievement of functional cure as a list of biomedical indices: undetectable viral load; no CD4 loss; lack of disease progression (and hence, no need for ART), and lack of HIV transmission risk [12]. HIV-positive patients fitting this description – lifelong -- after a curative intervention would be functionally cured^a^. In short, HIV cure research worthy of the name, independently of approach (ART initiation during acute infection, stem cell transplantation, gene therapy, ART intensification …) ultimately aims to contribute to a future sterilizing or functional cure. If these are the final goals of HIV cure research, the understanding of basic concepts such as sterilizing and functional cure should be as clear and unproblematic as possible.

In briefly exploring these notions, we will make four related claims: (1) definitions of sterilizing and functional cure will have to be carefully distinguished from the meanings given to ‘cure’ in everyday life as well as related medical notions, such as remission; (2) cure as understood in contemporary HIV cure literature belongs firmly in what is called the ‘biomedical model’. Given that this model has sometimes been criticized as reductive and inadequate in understanding disease (including HIV), the advantages and pitfalls of conceptualizing HIV cure from a purely biomedical perspective must be critically evaluated; (3) the distinction between sterilizing and functional cure is part of a larger and longstanding distinction (and tension) between what we will call absolute and modern conceptions of cure, particularly conspicuous in the context of cancer; (4) contemporary notions of HIV cure inherit some of the epistemic problems traditionally associated with cures for other health conditions. These four claims support the broader point that HIV cure concepts are works in progress, and raise a normative question: given the conceptual uncertainties, how should HIV research developments be talked about? Many cancer clinicians still shy away from talking about ‘cure’, even with cancers deemed curable [13]. Should we similarly avoid the ‘C word’ (i.e. cure) in the HIV context, and if so, why and when?

## Discussion

### The concept of HIV cure

#### The meanings of the commonsense notion of cure

The word ‘cure’ entered the English language in the 14^th^ Century, from the Latin *curare* (‘to take care of’). According to the Merriam-Webster Medical Dictionary, ‘cure’ can mean:

1. recovery from a disease (‘his cure was complete’); also: remission of signs or symptoms of a disease especially during a prolonged period of observation (‘a clinical cure’; ‘5-year cure of cancer’).
2. a drug, treatment, regimen, or other agency that cures a disease (‘quinine is a cure for malaria’).
3. a course or period of treatment; especially one designed to interrupt an addiction or compulsive habit or to improve general health (‘take a cure for alcoholism’; ‘an annual cure at a spa’).

#### The difference between an intervention as a cure and being cured

One can distinguish here between cure conceived as an *intervention* (‘drug, treatment, regime or other agency’; ‘course of treatment’) and cure as a *physical state of being in a person* brought about by that intervention (‘recovery’, ‘remission’). The proffered IAS and FDA definitions of sterilizing and functional cure are more *state of being* definitions than *intervention* definitions. This may be due to current uncertainty as to what approach, if any single one, will enact a cure in the ‘state of being’ sense. This distinction may be valuable in the context of research: it might help research participants avoid ‘fetishizing’ particular interventions as offering salvation from HIV by drawing attention to the desired future state of being cured.

#### HIV cure and the biomedical model

In 1977, Engel argued that the ‘biomedical model of disease’ was descriptively inaccurate and had negative consequences for the clinician-patient encounter [14]. By the ‘biomedical model of disease’, he meant the view that diseases are a derangement of underlying physical mechanisms and therefore can only be properly understood and managed at a physical (biological, virological, immunological, genetic) level. Engel argued instead that diseases – their classification, presentation, prevalence, distribution and so on -- are strongly shaped by social, cultural and psychological forces. In addition, he argued that ‘de-socializing’ disease could lead to inferior patient care, in the form of (for example) physicians discounting patient narratives of disease and relying too heavily on batteries of laboratory tests. Others have made a similar case in regard to mental health [9]. Engel believed that a broader, what he called a ‘biopsychosocial’ model of disease, would be more faithful to the phenomena and more supportive of a holistic approach to patient care. Since Engel’s writing, HIV has become a prime example of a serious infectious disease being increasingly approached by a broad, multidisciplinary ‘biopsychosocial’ model.

HIV cure definitions, however, are currently locked in the old biomedical framework. Sterilizing and functional definitions of HIV cure are all about physical states in the body. What would a biopsychosocial – patient-centered rather than disease-centered – conception of HIV cure involve? Would this involve changing the IAS/FDA definitions of HIV cure? Does their focus on physical states of the body render them inadequate, as Engel might argue? At least two options seem open: expand the notion of cure to incorporate social and psychological components of being cured, or reserve the concept of ‘cure’ for biological phenomena while embedding that construct in a larger biopsychosocial context. In regard to the latter, there are interesting questions about the relationship between cure and the related concept of healing: patients may be cured but not (yet) healed. On the physical level, the process of being cured of HIV may increase risks for other conditions, such as cancer or cardiovascular disease [10]. For example, a small population of people who are infected with HIV are able to control infection without medicines, such that their viral load is not detectable by standard tests. Although these so called “elite controllers” typically meet all criteria for the definition of functional cure, they are known to be at greater risk for chronic diseases than those without HIV infection. In addition, being cured of HIV biologically may be compatible with continued internalized or externalized stigma, i.e. persistent social and psychological effects of having had HIV. Wilson, Bladin and Saling report that being cured for a chronic condition involves a challenging process of adaptation (they refer to as the ‘burden of normality’) as the patient emerges from the ‘sick role’ and reconstructs his or her life activities, social relationships and self-identity [15]. Mullen talked of ‘seasons of survival’ to describe the emotional, social and medical adaptations required by continuing cancer survivorship over time [16]. Van Eys argued that cure has three components (biological, psychological and social), and to truly cure a patient requires all three [17]. It is tempting to conceive the biological form of cure as a necessary but not sufficient condition for healing, wholeness, and recovery. But this view does not represent the complex relationships between different dimensions of cure, for two reasons. First, healing may occur even in the absence of a physical cure. A person can come to terms with an illness, be healed in the sense of achieving a sense of ‘wholeness’, even though not cured on the biological level. Conversely, a person may be physically cured of a disease, but there may be residual sequelae of the disease or there may be side-effects of the means used in the process of cure. For example, HIV may cause neurological and cardiovascular defects that may persist even if the virus were to be eliminated. In such cases, even when a person no longer has HIV it may be hard to speak of him or her being ‘cured’.

#### Absolute and modern conceptions of cure

The distinction between sterilizing and functional HIV cures is part of a larger distinction between absolute and modern conceptions of cure, a distinction that pre-dates and transcends the context of HIV. According to the absolute conception, being cured of a health condition means you are rid of it. It is a state of being marked by finality. You had that health condition but now – generally thanks to some intervention – you do not have it anymore [18]^b^. The symptoms and the underlying causes of the condition no longer exist. The understanding of cures has likely been shaped by the idea of miraculous, religious cures enacted by prayer, divine intervention or touch by holy persons. Cases where the crippled (suddenly) walk or the blind (suddenly) see are the most dramatic transformative instances of an absolute cure. Curing appendicitis by surgery or pneumonia by antibiotics are secular, medical counterparts. The absolute conception continues to shape the contemporary understanding of cure to some extent. When an intervention does not seem to provide a cure in the absolute sense, uncertainty often arises as to whether we have warrant to speak about a *real* cure at all. In the scientific literature and popular press, there is a noteworthy tendency to use scare quotes (‘cure’) when talking about anything short of an absolute cure for HIV. The scare quotes pay tribute to the continued power of the notion of absolute cure over the imagination.

#### A modern conceptualization of cure

This is to be contrasted with a more modern conception of cure, which means that – thanks to some intervention – chances of disease resurgence have been significantly reduced. The definition by Easson and Russell [19], conceiving cure as disease-free survival over long duration, has been highly influential [19]^c^. The modern conception incorporates evidence-based uncertainty of outcome: you are cured where, on the best science available, there is a good chance that disease remission will continue. This conception is (for example) applied to certain cancers, hepatitis B, Epstein-Barr virus infection, toxoplasmosis and shingles – all instances where a pathogen is dormant and in selected populations can be reactivated and cause disease. The concept ‘cure’ in the construct of ‘cure-rate’ is synonymous with indefinite remission: a disease with a favorable cure rate (say, 80%) means the likelihood of disease reoccurrence with existing treatments is relatively low. Remission is transformed into cure (in this sense) on the basis of data about likely clinical outcomes over time. Where the absolute conception of cure is categorical, the modern conception is statistical.

Both cure conceptions currently circulate in biomedicine in complex ways. Physicians continue to hesitate to use the word ‘cure’, even when their patients have cancers with favorable cure rates [20]. They may avoid cure language in order to unduly raise hopes and avoid potential litigation, suggesting that some physicians believe their patients typically understand cure in an absolute sense. In a qualitative study of patient/physician interactions, Hamilton reports how while the standard goal of Hepatitis C therapy is *sustained virological response* (HCV RNA undetectable in serum for three months post-therapy), physicians frequently continued to speak of cure to HCV patients and invoked the absolute conception when doing so, particularly in initial consultations [21]. A possible explanation is that patients often do not, cannot, or do not want to understand cures for threatening diseases in terms of probabilities. Even if the question often cannot be answered, patients and families may want to know if they or their loved ones will be cured in an absolute sense. On the other hand, avoiding the word cure has its downsides: it may reduce patient confidence in the best intervention available, and negatively impact adherence and self-care. In order to anticipate issues with HIV cure, more empirical research is needed on how the concept ‘cure’ is used and understood in other medical domains where functional or sterilizing cures already exist.

#### Epistemic issues with sterilizing and functional cures

The definitions may seem straightforward, but challenges remain. With sterilizing cure, complete elimination of all remnants of HIV from the human body is an extremely high bar for cure. Elements of HIV may linger in the body after a successful curative intervention. This is currently the case with hepatitis C infection, where residual virus – whose clinical significance is unclear – has been detected by increasingly sensitive testing methods. It would seem better to alter (as some have done) the definition of sterilizing HIV cure to the elimination of *replication competent* proviruses in the body [22]. This may seem like a purely virological point, but the persistence of HIV debris (even if not ‘replication competent’) may have psychological and social import. If a cured person still has ‘neutralized’ HIV lingering in his or her body, will this have any impact on his or her social identity? Will he or she no longer be considered a person living with HIV/AIDS? We do not yet know. We also do not currently have diagnostics that can cheaply and readily detect the difference between replication competent HIV and non-replicating HIV debris. More definitive diagnostic tests increase the likelihood of a true sterilizing cure, but still will not guarantee it. The situation is exacerbated by a phenomenon already evident in cancer testing: increasingly sophisticated diagnostic tests that reveal traces of pathological conditions whose significance is unclear from clinical and existential perspectives. Lingering doubts that a sterilizing cure *really* has been achieved– in its absolute, categorical sense – may be very hard to shake.

The concept of functional cure is challenging in other ways. First, a problem well known in cancer circles: there will probably be no non-arbitrary way of determining when, after a curative intervention, the essential ingredients of undetectable viral load, no CD4 count loss, lack of disease progression, negligible transmission risk and treatment non-necessity add up to the state of being called ‘functionally cured of HIV.’ After how many years of HIV control without treatment (‘HIV remission’) will we know that the patient is functionally cured?^a^ Cancer physicians continue to order diagnostic tests for patients after even decades of disease remission (Miller et. al. [13]), highlighting their own lack of confidence. On the other hand, despite the uncertainties, being (probably) functionally cured will likely have health, economic, social and other advantages for some patients on ART: ‘cure purgatory’ may be superior to ‘treatment hell’. But considering the uncertainties and possible confusions surrounding sterilizing and functional cures, why talk about *cure* at all, especially now? Wouldn’t it be better to avoid it if possible, as some have recommended in relation to cancer? Why not replace it with (say) a state of being called ‘long term drug free remission’? Is talking about HIV cure at this early stage not just inaccurate or presumptive, but in some way *morally problematic*?

### The ethics of HIV cure talk

Why does language *morally* matter when it comes to HIV cure? One reason could stem from the notion of curative hope. Decades of clinical research have led to the development and refinement of antiretroviral treatment that has greatly benefited people living with HIV among those who have reliable access to it. However, life on HIV treatment has significant shortcomings. HIV-infected individuals bear the physical brunt of acting as host of the virus as well as the long-term effects of antiretroviral therapy. Many people on ART experience serious side effects, including headaches, nausea, vomiting, rashes, pancreatitis or liver failure. Over time, HIV takes a serious health toll even when the virus is under control, including increased risks of cardiovascular disease, many cancers, and dementia. There are also psychological effects and economic consequences of living with HIV and being on treatment. In short, persons living with HIV, including those on treatment, have a number of strong reasons to hope for a cure. Beyond wanting to be rid of a dangerous virus or the burdens of treatment regimes, it is also a matter of being liberated from the social identity associated with living with HIV, the interpersonal challenges that attach to that identity, and release from the fear of potentially transmitting HIV to offspring and loved ones. This is not to claim that gaining a ‘HIV-positive identity’ is only negative or that the loss of that identity would be unambiguously positive. Individuals may affect positive life changes as a result of becoming HIV infected, and the prospect of becoming a person no longer living with HIV may be seen as disadvantageous, e.g. loss of connection with the HIV community and its history or changes to medical benefits. Nevertheless, these complex identity concerns are unlikely to completely dampen desires for cure.

With this powerful curative hope in the background, how might cure language ‘do wrong’? The most obvious way is falsehoods through unsubstantiated claims of HIV cure. Many such claims have been made worldwide over the last decades, and this violates ethical norms of veracity and non-maleficence. A study in Tanzania suggests that when traditional healers claim they can cure HIV, there are negative effects on ART adherence among their HIV-positive clients [23]. But misunderstandings are likely to develop in subtler and less intentional ways. Traditional media, Internet news outlets and online social networks use language sometimes suggest a (safe, effective, scalable, affordable) cure may have been discovered or is just over the horizon. These claims could arguably do psychological harm to persons living with HIV by unduly raising and dashing hopes, and the generation of false beliefs might have other negative effects on behavior. Is this ‘harms due to false belief via cure language’ scenario plausible? Some considerations in support of this scenario include: the media’s vested economic interest in (to a greater or lesser extent) ‘sensationalizing’ HIV cure research; that even if what is accurately reported is a potential functional cure only applicable to a small minority of HIV-positive persons, the grip of the absolute conception of cure on the public imagination may lead some to misunderstand current HIV research advances. To what extent is this a problem? This is another area for future social science research.

In the short term, concern about ‘harms due to false belief via cure language’ is likely to center on informed consent to HIV cure research. False beliefs on the part of prospective research participants may hinder the process of obtaining valid informed consent in HIV cure studies. Other forms of misconception, also involving false beliefs, are already recognized in research. In treatment studies, the *therapeutic misconception* is the well-documented phenomenon of research participants mistakenly believing that they stand personally to benefit from the medical intervention being tested [24]. In prevention studies, the *prophylactic misconception* consists in research participants mistakenly believing they will be protected by the preventive intervention being tested (such as a candidate HIV vaccine), and possibly taking greater health risks as a consequence. A *curative misconception* in HIV research would involve HIV-positive research participants falsely believing that they stand to be cured by the tested intervention. Such beliefs could cloud the participant’s understanding of what the research involves, unduly influence his or her willingness to participate in cure studies, distort risk perception, and fuel risk behavior. As antidote to curative misconception, some call for proof-of-concept HIV cure studies to be called ‘experiments’ to emphasis the risks and play down the possibility of individual benefit [25]. Besides the effects of potential misconceptions, talk of HIV cure might impact on ART treatment acceptance. Ingrid Katz has shown that large populations of persons living with HIV already refuse antiretroviral treatment [26]. Future research will need to focus on whether people refuse ART because they believe a better, ‘definitive’ solution to HIV infection could be on the horizon.

We have raised some ethical worries about the use of the concept ‘cure’. These should not be overblown:

1. *Whether hope for HIV cure is harmful is a complicated issue that requires future empirical investigation and ethical analysis.* Worries about ‘false hope’ may involve a misunderstanding of hope, and underestimate the potentially positive effects of hope as a psychological disposition and orientation to the world. In the context of HIV research, curative hopes are powerful desires for an outcome believed to be good but whose fulfillment is uncertain. If so, stimulating desires for a HIV cure by using cure language does not seem wrong unless false beliefs are involved in the process. Following Horng and Grady, one should distinguish between curative misconception, curative misestimation, and curative optimism. If the beliefs involved in hope are true, i.e. if HIV-positive persons know what cure would (and would not) involve and have realistic expectations of what current research can produce, then the use of cure talk would not raise false hope and destructive optimism. However, cure talk might just be keeping hope of a better future alive, as the dream of cure has done for HIV-positive persons long before the recent clinical discoveries. Quantitative and qualitative psychological research has indicated the beneficial effects of maintaining a positive mindset. Although further study is needed, positive attitudes towards the future curability of HIV may help in improving quality of life for persons living with the virus. Whether HIV cure talk will and to what extent lead to broader improvement in health seeking behavior (such as regular HIV testing) remains to be seen: beliefs about curability may hamper HIV prevention efforts, as treatability has sometimes done [27]. It is also possible, as Miller et. al. suggest in the context of oncology, that patients who believe they are cured may in fact neglect their care, and future physicians may also regard the continuing care of ‘cured patients’ as a lower priority relative to other pressing health needs.
2. *The ethical question is not whether to use the concept of HIV cure, but how*, *when and for what purpose*. There is something of a split between what terms many scientists/clinicians are comfortable with (remission, sustained virological suppression, viral suppression off treatment) and what ‘non-experts’ would prefer to use [28]. Some argue that in clinical contexts, ‘HIV remission’ would be a better term than ‘HIV cure’, because remission avoids ambiguities associated with cure, is relatively familiar to non-experts, and sends the message to patients that monitoring and self-care remain important [29]. Outside the clinical context, there may be legitimate and responsible uses of the concept of ‘HIV cure’. Advocacy groups, for example, will undoubtedly deploy ‘cure’ when raising community awareness and engaging the broader public. Similar to cancer initiatives, expressions like the ‘race for a cure’ are important means of rallying and mobilizing groups around a common cause. The same motivational effects might not be achieved by evoking continued viral suppression. It is also possible – another area of study – that explicitly and repeatedly linking ‘HIV’ and ‘cure’ will help chip away at the stigma associated with the virus, and have other positive knock-on effects in the HIV prevention and treatment worlds. This too is unsure: labeling successfully treated patients as ‘cured’ of HIV may increase the stigma of those treated but unable to achieve a cure. Predictably, the language of HIV cure will have intended and unintended implications, positive and negative. It will be important for social scientists and ethicists to anticipate and track these as researchers get closer to durably controlling, and hopefully eliminating, HIV.

## Summary

Promising clinical developments have raised hopes for a cure for HIV among researchers, clinicians, patients and the general public. Efforts to gain conceptual clarity about the meaning of ‘cure’ are not mere semantic exercises, for they can elucidate the goals of HIV research and future clinical care. In this paper, we examine some of the meanings of cure, as well as their assumptions and historical precedents. We argue that attention to language ethically matters in this context, and identify some advantages as well as potential pitfalls of how different HIV/AIDS stakeholders may employ the concept of cure. While concepts other than cure (such as remission or viral suppression) may be appropriate in clinical contexts, use of the word cure may be justified for other important purposes in the struggle against HIV/AIDS.

## Endnotes

^a^According to the IAS definition of functional HIV cure as *lifelong* control of virus in the absence of ART, it seems functional cure can only be established at the end of a person’s life. In this sense, not even the Berlin Patient can be said to be functionally cured yet. Definitions of functional cure will likely change as more becomes known about impact of curative interventions on patient outcomes.

^b^As Hillon et. al. put it: “The term ‘cure’ is defined, *inter alia*, as the end of a medical condition, a substance or procedure that ends the condition, or the state of being healed, or cured. This is therefore an absolute concept, and inherent in that is the permanent (and complete) end to a specific instance of the disease” [18].

^c^“… cure of a disease is taken to connote that in time – probably a decade or two after treatment – there remains a group of disease free survivors whose annual death rate from all causes is similar to that of a normal population group of the same sex and age distribution” [19].
